# Walking through trimesters: a quantitative assessment of step counts before and during pregnancy and their impact on pregnancy outcomes

**DOI:** 10.1007/s00404-026-08407-1

**Published:** 2026-04-28

**Authors:** Michal Ovadia, Noga Nattiv, Rivka Podorovsky Banker, Yair Daykan, Ron Schonman, Tal Biron Shental, Michal Kovo, Yael Yagur, Omer Weitzner

**Affiliations:** 1 Department of Obstetrics and Gynecology, Meir Medical Center, 4428164 Kfar Saba, Israel; 2 School of Medicine, Gray Faculty of Medical and Health Sciences, Tel Aviv University, Tel Aviv, Israel; 3 Assaf Harofeh Medical Center, Tel Aviv, Israel

**Keywords:** Physical activity, Step count, Pregnancy, Delivery outcomes, Delivery complications

## Abstract

**Objective:**

To examine walking behavior during pregnancy and its association with obstetric and neonatal outcomes.

**Methods:**

This retrospective cohort study included pregnant patients who delivered between 2020 and 2024 at a tertiary medical center. Daily step counts were recorded via the Health Auto Expert mobile application from three months before conception until admission to the labor ward. Pregnancy and delivery data were obtained from medical records. The primary outcome was a composite of delivery complications by step-count category: Above-Average and Below-Average, based on the cohort mean. Secondary outcomes included postpartum complications, mode of delivery, and neonatal outcomes.

**Results:**

The mean daily step count during pregnancy was 2539, declining progressively with gestation. Of 176 patients, 71 (40%) were Above-Average and 105 (60%) Below-Average. The Above-Average group had higher pre-pregnancy activity (4541.7 vs 2330.3 steps/day, *p* < 0.001) and higher maternal age. No significant differences were found in composite delivery or postpartum complications. Vacuum extraction was more frequent in the Above-Average group (15.5% vs 3.8%, *p* = 0.014), though not significant after adjustment. Median neonatal birth-weight percentile was 54.5, lower in the Above-Average group (44.0 [25.0–71.0] vs 60.0 [38.0–78.0], *p* = 0.024), without significance in multivariable analysis.

**Conclusions:**

Walking patterns declined across pregnancy. Step count was not associated with delivery complications or adverse maternal or neonatal outcomes. Walking during pregnancy appears safe and was not associated with adverse maternal or neonatal outcomes in this predominantly sedentary cohort of low-risk pregnancies.

## What does this study adds to the clinical work

**Table:** 

This study shows that objectively measured daily step counts throughout pregnancy are not associated with adverse maternal or neonatal outcomes. These findings support that routine ambulatory activity is safe in low-risk pregnancies and may reassure clinicians and patients.	

## Introduction

Research across many platforms has shown that regular physical activity is one of the most important actions people can take to improve their health [1]. Pregnant or postpartum patients also benefit from regular physical activity [2], but a population-based survey in the United States determined that a substantial proportion (60%) of pregnant patients do not engage in either moderate or vigorous leisure physical activity [3, 4].

Most studies have focused on specific structured exercises, such as Pilates or supervised activities like aerobic or water-based workouts, which require patients to allocate dedicated time for participation [5]. In contrast, data on the effects of walking, as a natural part of daily life on pregnancy outcomes, remain sparse [6].

Activity levels below 5,000 steps per day are considered sedentary. A randomized trial in pregnant patients with obesity and early gestational diabetes advised to take ≥5,000 steps daily found that pedometer monitoring did not significantly affect metabolic control or weight gain but reduced macrosomia, while exceeding this threshold improved weight loss and obstetric and neonatal outcomes [7]. A recent randomized trial found that encouraging ≥ 2,000 steps daily in patients with preterm pre-labor rupture of membranes prolonged latency, reduced neonatal complications, and improved maternal mental health [8].

As pregnant patients walk not only for exercise but also during daily activities such as work, household tasks, and general mobility, this study focused on step counts throughout pregnancy to assess walking behavior and its associations with delivery outcomes and postpartum complications. However, research investigating the impact of such routine physical activity on pregnancy outcomes, specifically in low-risk women, remains sparse. Therefore, our study aimed to address this gap by investigating the association between daily step counts and pregnancy and delivery outcomes in this population.

## Methods

### Study population

This retrospective cohort study was conducted at a tertiary academic hospital and included pregnant patients who delivered between 2020 and 2023. Daily step counts were obtained from participants’ personal iPhone *Health* applications. Eligible women were aged 18–45, had low-risk pregnancies, carried their iPhone for over 80% of their mobility time, and had no contraindications to physical activity. Exclusion criteria included multiple gestations, fetal anomalies, medical conditions limiting activity, prior preterm birth, or inconsistent phone use. The mean daily step count during pregnancy was calculated for each participant. For exploratory comparison, participants were categorized into two groups according to whether their mean daily step count was above or below the cohort mean. This categorization was intended to reflect relative differences in ambulatory activity within the cohort.

### Outcomes

The primary outcome was composite delivery complications based on the number of steps during pregnancy) (induction of labor, intrapartum fever, vacuum extraction, cesarean section, non-reassuring fetal heart rate, and failed induction). Secondary outcomes included: (1) composite postpartum complications (postpartum hemorrhage, lysis, decreased hemoglobin level, need for blood transfusion, and surgical scar infection); (2) mode of delivery (vaginal, instrumental, or cesarean delivery); (3) neonatal outcomes including birthweight and Apgar scores. Potential confounding factors considered included maternal age, gravidity, parity, BMI, comorbidities, smoking status.

### Study protocol

The participants were recruited from the maternity ward 6 and up to 96 h after delivery. After agreeing to participate in the study, providing informed consent, and screening for inclusion criteria, the participants were asked to download the Health Auto Expert mobile application on their iPhones. The app automatically tracked and recorded their step counts, and the participants were instructed to select the relevant period from 3 months before their pregnancy through their delivery. These step count data were then emailed to the study team. Medical background, pregnancy and delivery outcome data were collected from patients’ medical records.

### Ethics

The study followed the Declaration of Helsinki, approved by the Institutional Review Board of Meir Medical Center (MMC 0031-20). All participants gave informed consent for use of their step count and medical data. Patient confidentiality was maintained throughout.

### Statistical analysis

Patient characteristics were compared between the groups using Student’s t-test for continuous variables and the Chi-square or Fisher’s Exact Test for categorical variables. Continuous and ordinal variables were compared between groups using independent samples t-test or Mann–Whitney test. Data distribution was assessed prior to analysis. Variables with non-normal distributions, including daily step counts, were analyzed using the Mann–Whitney U test and are presented as medians with interquartile ranges. Data are presented as number and percentage for categorical variables and as mean and standard deviation (SD) for continuous variables. A logistic regression model was constructed, with Delivery Complications serving as the dependent variable. Independent variables included step count group as the primary predictor, while potential confounders such as maternal age and BMI were added to the model. A second model for neonatal outcomes used the confounders—baby weight and Dolberg score (birthweight percentiles based on gestational age at delivery using standardized Israeli population-based reference charts)—that were added to the model [9].

All statistical tests were two sided and p < 0.05 was considered statistically significant. Data were analyzed by SPSS statistical analysis software v23.0 (IBM Inc., USA).

Because no prior studies have reported the incidence of an identical composite delivery complication outcome by step-count categories, the expected effect size was guided by studies linking physical activity in pregnancy with labor and delivery outcomes [10, 11]. These reports describe clinically meaningful absolute differences of approximately 8–10% between higher- and lower-activity groups.

Based on this range, a cohort of approximately 170–200 women provides an estimated 70–80% power to detect such differences at α = 0.05. The sample size was sufficient to detect moderate effect sizes reported in prior studies. Our final sample included 176 women, which falls within this range and is therefore adequately powered to evaluate the association between step counts and the composite delivery outcome.

## Results

### Walking patterns during pregnancy

During the study period, 176 patients were enrolled. The mean daily step count was 2539 (range 128–6700). Participants were divided into two groups based on this threshold: below-average and above-average step counts. Of the participants, 105 (60%) averaged fewer than 2539 steps per day and were classified as the “Below Average” group, while 71 (40%) averaged 2539 steps or more and were classified as the “Above Average” group.

**Fig. 1:**
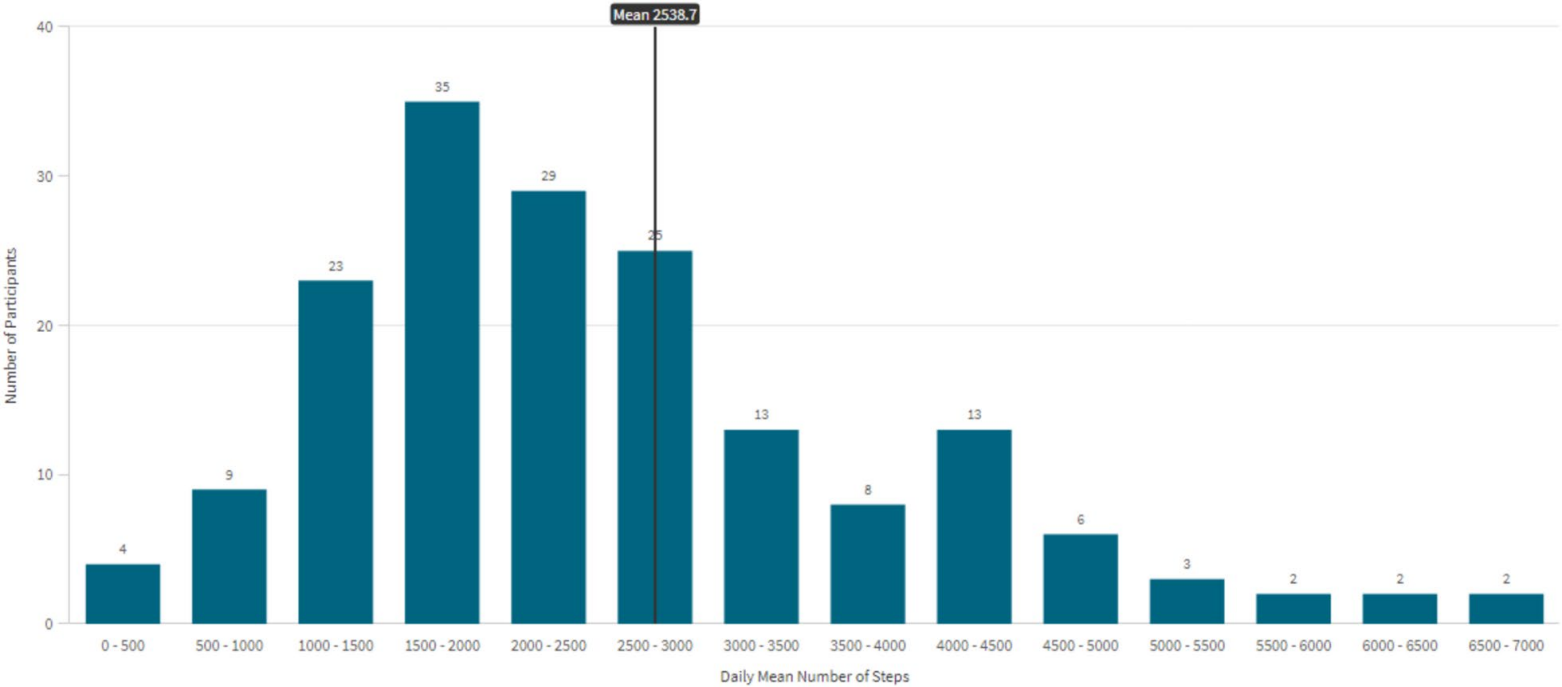
Distribution of daily mean steps among study population (*n* = 176)

Figure 1 shows the distribution of daily mean steps, with most participants recording 1000–3000 steps per day. The vertical line represents the overall mean of 2539 steps per day. The x-axis shows step count ranges in intervals of 500 steps, and the y-axis shows the number of participants in each range.

Table 1 presents walking patterns across trimesters, showing a steady decline in activity as pregnancy progressed.

**Table 1:** Walking patterns across pregnancy trimesters

Period	Daily steps	Percentage change from pre-pregnancy
Pre-pregnancy	3287 ± 2390	–
1st trimester (0–14 weeks)	2804 ± 2034	– 14.7%
2nd trimester (14–26 weeks)	2626 ± 1922	– 20.1%
3rd trimester (26–40 weeks)	2298 ± 1761	– 30.1%
Average steps during pregnancy	2539 ± 1916	– 22.8%

Data are shown as number of steps per a day, mean ± standard deviation

### Demographic and baseline characteristics

The mean age of participants was 30.6 ± 5.8 years. As shown in Table 2, the Above-Average group had a higher mean maternal age than the Below-Average group (31.7 ± 5.7 vs. 29.9 ± 5.8 years; *p* = 0.045). Pre-pregnancy physical activity, measured as mean daily steps, was significantly higher in the Above-Average group (4541.7 ± 1630.8 vs. 2330.3 ± 1021.3; *p* < 0.001). BMI data were available for 92 participants. Among those with available data, 62% had BMI < 24.9, 25% had BMI between 25 and 29.9, and 13% had BMI ≥ 30, with no significant differences between groups (*p* = 0.156). Smoking status, gravidity, parity, and pre-gestational diabetes also did not differ significantly between groups.

**Table 2:** Demographic and baseline characteristics of study population

Characteristic	Above average (*n* = 71)	Below average (*n* = 105)	*P*-value
Age (years)	31.7 ± 5.7	29.9 ± 5.8	0.05
BMI (kg/m^2^)			
BMI < 24.9	27 (73.0%)	30 (54.5%)	0.19
BMI 25–29.9	6 (16.2%)	17 (30.9%)	0.19
BMI > 30	4 (10.8%)	8 (14.5%)	0.19
Smoking	5 (4.8%)	5 (4.9%)	0.74
Mean steps/day in pre-pregnancy	4541.7 ± 1630.8	2330.3 ± 1021.3	< 0.001
Gravidity	2.0 [1.0,4.0]	2.0 [1.0,3.0]	0.66
Pre-gestational diabetes	1 (1.4%)	1 (1.0%)	1.00

Data are shown as number (%), mean ± standard deviation or median (range), as appropriate

*BMI* body mass index (kg/m^2^)

BMI data were available for 92 participants

### Pregnancy outcomes and complications

Table 3 compares pregnancy outcomes and complications between the study groups.

**Table 3:** Pregnancy outcomes and complications between the study groups

Characteristic	Above average (*n* = 71)	Below average (*n*= 105)	*P*-value
*Gestational diabetes	9 (12.7%)	17 (16.2%)	0.70
Hypertension	8 (11.3%)	15 (14.3%)	0.71
Gestational age at delivery	39.2 ± 1.4	39.0 ± 1.3	0.57
Delivery before 39 weeks	21 (29.6%)	38 (36.2%)	0.35
Delivery ≥ 41 weeks	8 (11.3%)	5 (4.9%)	0.35
Length of maternal hospitalization	3.0 [2.0,3.0]	3.0 [2.0,4.0]	0.07

Data are shown as number (%), mean ± standard deviation or median (range), as appropriate

*Gestational diabetes (includes GDMA1 and GDMA2)

Gestational diabetes occurred in 26 (14.8%) participants overall, with a non-significant trend toward lower incidence in the Above-Average group (9 [12.7%] vs. 17 [16.2%]; *p* = 0.669). Hypertension rates were similar between groups (8 [11.3%] vs. 15 [14.3%]; *p* = 0.71). Preterm delivery (< 39 weeks) occurred in 59 (33.9%) participants, 21 (29.6%) in the Above-Average group and 38 (36.2%) in the Below-Average group (*p* = 0.353). Deliveries beyond 41 weeks were more frequent in the Above-Average group, though not statistically significant (8 [11.3%] vs. 5 [4.9%]; *p* = 0.198).

### Delivery and postpartum outcomes

Table 4 compares delivery and postpartum outcomes between the study groups.

**Table 4:** Labor and postpartum outcomes between the study groups

Characteristic	Above average (*n* = 71)	Below average (*n* = 105)	*p*-value
Induction of labor	27 (38.0%)	48 (45.7%)	0.33
Fever during labor	2 (2.9%)	3 (2.9%)	1.00
Vaginal delivery	45 (63.4%)	76 (72.4%)	0.27
Vacuum extraction	11 (15.5%)	4 (3.8%)	**0.01**
Cesarean delivery and indications	15 (21.1%)	23 (21.9%)	1.00
NRFHR^$^	4 (5.6%)	10 (9.5%)	0.51
Failed induction	3 (4.2%)	1 (1.0%)	0.30
Postpartum hemorrhage	3 (4.2%)	5 (4.8%)	1.00
Manual revision of placenta	4 (5.6%)	3 (2.9%)	0.44
Hemoglobin level after delivery, g/dL	10.8 ± 1.5	10.8 ± 1.7	0.98
Delta hemoglobin (before-after), g/dL	-1.6 ± 1.1	-1.4 ± 1.4	0.57
Postpartum fever	3 (4.2%)	2 (1.9%)	0.40
Blood transfusion	1 (1.4%)	4 (3.9%)	0.65
Surgical scar infection	2 (2.8%)		0.17
Composite delivery complications*	32 (45.1%)	36 (34.3%)	0.20
Composite postpartum complications^^^	3 (4.2%)	7 (6.7%)	0.74

Data are shown as number (%), mean ± standard deviation or median (range), as appropriate

^$^NRFHR: non reassuring fetal heart rate

*Composite delivery complications included induction of labor, intrapartum fever, vacuum extraction, cesarean section, non-reassuring fetal heart rate episodes and failed induction

^Composite postpartum complications included postpartum hemorrhage, lysis, decreased hemoglobin level, need for blood transfusion and surgical scar infection

Induction of labor was required in 75 (43.1%) participants overall, occurring in 27 (38.0%) of the Above-Average group and 48 (45.7%) of the Below-Average group, with no significant difference (*p* = 0.334). The Above-Average group had a significantly higher rate of vacuum-assisted deliveries (11 [15.5%] vs. 4 [3.8%]; *p* = 0.014), while rates of vaginal and cesarean deliveries did not differ. Multivariate logistic regression showed that belonging to the Above-Average group was associated with higher odds of vacuum-assisted delivery (OR 3.86, 95% CI 0.71–21.09; *p* = 0.09), though not statistically significant after adjustment. No significant differences were observed between groups in rates of vaginal or cesarean delivery, non-reassuring fetal heart rate, failed induction, or postpartum outcomes. Composite delivery complications were more frequent in the Above-Average group (32 [45.1%] vs. 36 [34.3%]; *p* = 0.2), but this was not statistically significant, and composite postpartum complications also showed no significant difference.

### Neonatal outcomes

Table 5 presents neonatal outcomes. The mean birth weight was 3206 ± 444.7 g overall, 3124 ± 478.1 g in the Above-Average group, and 3262 ± 413.1 g in the Below-Average group (*p* = 0.051). The median birth weight percentile was 54.5 overall, significantly lower in the Above-Average group (44.0 [25.0–71.0]) than in the Below-Average group (60.0 [38.0–78.0]; *p* = 0.024). Birthweight distributions and birthweight percentiles were similar across step-count groups, with no significant differences between them. Multivariate logistic regression indicated that being in the Above-Average group was associated with slightly higher odds of a lower Apgar score (OR 0.94, 95% CI 0.87–1.03; *p* = 0.18) after adjusting for maternal age, BMI, and baby weight, though this was not statistically significant.

**Table 5:** Neonatal outcomes between study groups

Characteristic	Overall (*n* = 176)	Above average (*n* = 71)	Below average (*n* = 103)	*P*-value
Birth weight, g	3,206 ± 444.7	3,124 ± 478.1	3,262 ± 413.1	0.05
Birth weight < 2500 g	15 (8.6%)	9 (12.7%)	6 (5.8%)	0.18
Birth weight 2500–4000 g	154 (88.5%)	59 (83.1%)	95 (92.2%)	0.18
Birth weight > 4000 g	5 (2.9%)	3 (4.2%)	2 (1.9%)	0.18
Birth weight percentile	54.5	44.0 [25.0,71.0]	60.0	**0.02**
BW percentile < 10	[33.0,76.8]	8 (11.3%)	[38.0,78.0]	0.11
BW percentile 10–89	12 (6.9%)	55 (77.5%)	4 (3.9%)	0.11
BW percentile > = 90	146 (83.9%)	8 (11.3%)	91 (88.3%)	0.11
Apgar score at 5 min < 7	4 (2.3%)	1 (1.4%)	8 (7.8%)	0.65
			3 (2.9%)	

Data are shown as number (%), mean ± standard deviation or median (range), as appropriate

## Discussion

This study examined the impact of maternal step counts during pregnancy on induction of labor, antenatal complications, delivery outcomes, and neonatal outcomes. No significant associations were found between step count during pregnancy and delivery or postpartum complications. Importantly, given that the study population demonstrated relatively low baseline activity levels, our findings should be interpreted as reflecting the effect of spontaneous daily ambulation rather than formal exercise interventions**.**

As far as we know, there is no current literature that investigates evidence linking the number of steps per day during the entire pregnancy to obstetric outcomes. The GESTAFIT Project measured steps per day using triaxial accelerometers but only for 7 consecutive days during the second trimester. However, they claimed that based on these 7-day measurements, increased physical activity and decreased sedentary time during pregnancy were associated with improved neonatal birth markers measured by arterial cord blood oxygen saturation, with no significant differences being found between vaginal and cesarean deliveries [12]. A prospective cohort study assessed physical activity using the Kaiser Physical Activity Survey in each trimester, which evaluated housework/caregiving, active living habits, sports, and occupation, rather than objective measurements. Rates of prolonged second stage, cesarean, operative and vaginal delivery, and perineal lacerations were similar between patients with higher and lower physical activity levels [11].

Adamczak et al. specifically examined pregnant patients with diabetes and obesity who monitored their physical activity with pedometers to analyze the impact on their metabolic control, weight gain and obstetric outcomes [7]. In contrast to the previously described studies, our study tracks the total number of steps taken throughout pregnancy in a low-risk population. Most studies evaluated physical activity at varying intensities, whereas our study specifically focused on step counts, providing an alternative method for monitoring movement patterns among pregnant patients.

Step count may not fully capture the type or intensity of physical activity required to influence labor outcomes, as it primarily reflects general movement, not structured or purposeful physical activity.

The step-count levels observed in this cohort were substantially lower than thresholds previously associated with obstetric or metabolic benefit, and therefore our findings should not be interpreted as contradicting the established benefits of moderate-intensity physical activity. Nevertheless, we observed a non-significant trend suggesting a higher incidence of composite delivery complications in the group with above-average daily steps, which might indicate that a larger sample size could reveal a clearer association. Additionally, actual activity levels could have been underestimated if participants did not consistently carry their phones during exercise. Future studies with larger cohorts and more precise activity tracking methods are recommended.

In our study, no significant differences were found between average steps per day and the mode of delivery. One finding was the significantly higher rate of vacuum extraction in the Above-Average group in univariate analysis. To better understand this finding, a logistic regression model was constructed, with no significance. Studies found that prenatal exercise is associated with a reduction in the odds of instrumental deliveries [13–15]. The discrepancy between our findings and those in the literature may stem from the fact that our study assessed step count exclusively, rather than overall physical activity. Moreover, the wide confidence interval suggests limited precision in our estimate, likely due to the relatively small sample size.

Evaluation of neonatal outcomes revealed that birth weight might be associated with the number of steps. In the Above-Average steps group, birth weight percentiles were lower compared to the Below-Average group (44.0 vs 60.0; respectively, *p* = 0.024). However, after adjusting for confounders this association did not reach statistical significance.

Studies directly linking daily step count to neonatal birth weight are limited. A meta-analysis of 28 randomized controlled trials found that prenatal exercise reduced the likelihood of delivering large for gestational age infants and lowered mean birth weight, without increasing the risk of small for gestational age newborns [16]. Exercise interventions in women of normal weight and those with overweight or obesity similarly showed no increased risk of growth restriction [17–19]. However, Hopkins et al. [20] reported lower birth weights among infants of mothers who exercised throughout pregnancy, while another large meta-analysis found no significant differences in birth weight or rates of low birth weight between exercise and control groups [21].

In our study, infants born to more physically active mothers had lower birth weight percentiles but remained within the normal range, suggesting appropriate fetal growth. This may also reflect potential long-term metabolic benefits, as lower-normal birth weights have been associated with reduced risks of obesity and metabolic disorders later in life [17]. Additionally, maternal physical activity may influence long-term adiposity risk; McDonald et al [22] found that prenatal aerobic exercise (150 min per week) did not affect fetal morphometrics at 36 weeks but reduced infant body fat and skinfold thickness at one month, indicating early benefits for adiposity regulation [23].

Our study has several strengths. Using step counts as an objective measure of physical activity provided a concrete, quantifiable assessment and minimized recall bias associated with self-reported data. This method captured diverse daily movements, including childcare and household activities, often excluded from traditional definitions of exercise. The study evaluated a wide range of maternal and neonatal outcomes, offering a comprehensive view of the potential effects of physical activity. Continuous monitoring from pre-pregnancy through all trimesters allowed detailed assessment of changes over time. The inclusion of a homogeneous population without pre-existing conditions reduced confounding and strengthened the validity of the findings. Overall, our results support that walking during pregnancy is safe and may be recommended as a beneficial form of physical activity for pregnant women.

However, this study has several limitations. Its retrospective design carries inherent risks of bias in data collection and analysis. Although step counts provide an objective measure, accuracy may have been affected by dependence on smartphone use, as steps taken without the device or non-step activities (e.g., swimming, cycling) were likely underreported. Despite adequate power for major outcomes, smaller differences, particularly for gestational diabetes or hypertensive disorders, may have been underpowered. Additionally, step counts were categorized into above- and below-average groups based on the cohort mean. This approach, while facilitating descriptive comparison between relatively more and less active participants, may reduce statistical power and does not represent a clinically validated activity threshold. Therefore, the findings should be interpreted as reflecting relative differences in ambulatory activity within this predominantly sedentary population. The absence of significant group differences contrasts with previous studies reporting a protective effect of physical activity, possibly due to sample size, population characteristics, or the categorization of activity levels. These values represent predominantly sedentary activity and may differ across populations; however, they accurately characterize real-world activity levels within our cohort. This study reflects unmodified real-world ambulatory activity, without educational or motivational interventions, which limits comparability with structured exercise trials but strengthens ecological validity. In addition, BMI were not available for all participants due to the retrospective nature of the study. Multivariable regression analyses were therefore performed using available cases for the variables included in the model, which may have reduced the effective sample size. Finally, the exclusive use of step counts may not capture qualitative aspects of walking—such as intensity or duration—that could influence outcomes and explain discrepancies with prior research.

Our findings suggest that routine daily walking was not associated with adverse maternal or neonatal outcomes in this predominantly sedentary cohort. These results provide reassurance regarding the safety of habitual daily ambulation in low-risk pregnancies. Walking is accessible, low risk, and may support broader maternal health benefits such as chronic disease prevention, improved mood, and overall well-being. Although most delivery and postpartum outcomes were similar between activity groups, step counts provided meaningful insight into routine physical activity patterns during pregnancy. Overall, maintaining daily movement appears safe, with no evidence of harm. The clinical implication of this study is reassurance regarding the safety of routine daily walking within a predominantly sedentary population, rather than evidence for benefit from moderate or high-intensity physical activity. Larger, more diverse studies with long-term follow-up are needed to confirm these results and clarify the potential effects of daily walking on pregnancy complications and outcomes for both mother and child.

## Data Availability

Anonymized data are stored on secure hospital servers and cannot be publicly shared. Access may be granted upon reasonable request and institutional approval.

## References

[1] Clapp J, Capeless E (1991) Neonatal morphometrics after endurance exercise during pregnancy. Int J Gynaecol Obstet 36(4):348–348. 10.1016/0020-7292(91)90505-Y 10.1016/0002-9378(90)90754-u 2256486

[2] Bull FC, Al-Ansari SS, Biddle S, Borodulin K, Buman MP, Cardon G et al (2020) World Health Organization 2020 Guidelines on physical activity and sedentary behaviour. Br J Sports Med 54(24):1451–1462. 10.1136/bjsports-2020-102955 33239350 10.1136/bjsports-2020-102955 PMC7719906

[3] ACOG Committee (2020) Physical activity and exercise during pregnancy and the postpartum period: ACOG Committee Opinion Summary, Number 804. Obstet Gynecol 135(4):991–3. 10.1097/AOG.0000000000003773 10.1097/AOG.0000000000003773 32217974

[4] Ruchat SM, Mottola MF, Skow RJ, Nagpal TS, Meah VL, James M et al (2018) Effectiveness of exercise interventions in the prevention of excessive gestational weight gain and postpartum weight retention: a systematic review and meta-analysis. Br J Sports Med 52(21):1347–1356. 10.1136/bjsports-2018-099399 30337461 10.1136/bjsports-2018-099399

[5] Price BB, Amini SB, Kappeler K (2012) Exercise in pregnancy: effect on fitness and obstetric outcomes-a randomized trial. Med Sci Sports Exerc 44(12):2263–2269. 10.1249/MSS.0b013e318267ad67 22843114 10.1249/MSS.0b013e318267ad67

[6] Evenson KR, Mottola MF, Artal R (2019) Review of recent physical activity guidelines during pregnancy to facilitate advice by health care providers. Obstet Gynecol Surv 74(8):481–489. 10.1097/OGX.0000000000000693 31418450 10.1097/OGX.0000000000000693

[7] Adamczak L, Mantaj U, Sibiak R, Gutaj P, Wender-Ozegowska E (2024) Physical activity, gestational weight gain in obese patients with early gestational diabetes and the perinatal outcome - a randomised-controlled trial. BMC Pregnancy Childbirth 24(1):104. 10.1186/s12884-024-06296-3 38308265 10.1186/s12884-024-06296-3 PMC10836025

[8] Pineles BL, Vial M, Castro T, Ghorayeb T, Ajishegiri O, Sadek S et al (2024) Ambulation for latency during expectant management of preterm prelabor rupture of membranes: a randomized controlled trial (AMBLE). Am J Obstet Gynecol MFM 6(1):101218. 10.1016/j.ajogmf.2023.101218 37944668 10.1016/j.ajogmf.2023.101218

[9] Rubin L, Haklai Z, Dollberg S, Zimmerman D, Gordon ES (2022) Improved method for revising the Israel birthweight references. J Perinat Med 50(7):977–984. 10.1515/jpm-2021-0401 35585723 10.1515/jpm-2021-0401

[10] Baena‐García L, de la Flor‐Alemany M, Coll‐Risco I, Reoyo OR, Aranda P, Aparicio VA (2023) A concurrent prenatal exercise program increases neonatal and placental weight and shortens labor: the GESTAFIT project. Scand J Med Sci Sports 33(4):465–474. 10.1111/sms.14298 36578199 10.1111/sms.14298

[11] Watkins VY, O’Donnell CM, Perez M, Zhao P, England S, Carter EB et al (2021) The impact of physical activity during pregnancy on labor and delivery. Am J Obstet Gynecol 225(4):437.e1-437.e8. 10.1016/j.ajog.2021.05.036 34081895 10.1016/j.ajog.2021.05.036 PMC10564562

[12] Baena‐García L, Ocón‐Hernández O, Acosta‐Manzano P, Coll‐Risco I, Borges‐Cosic M, Romero‐Gallardo L et al (2019) Association of sedentary time and physical activity during pregnancy with maternal and neonatal birth outcomes. The GESTAFIT Project. Scand J Med Sci Sports 29(3):407–414. 10.1111/sms.13337 30450596 10.1111/sms.13337

[13] Davenport MH, Ruchat SM, Sobierajski F, Poitras VJ, Gray CE, Yoo C et al (2019) Impact of prenatal exercise on maternal harms, labour and delivery outcomes: a systematic review and meta-analysis. Br J Sports Med 53(2):99–107. 10.1136/bjsports-2018-099821 30337349 10.1136/bjsports-2018-099821

[14] Takami M, Tsuchida A, Takamori A, Aoki S, Ito M, Kigawa M et al (2018) Effects of physical activity during pregnancy on preterm delivery and mode of delivery: The Japan Environment and Children’s Study, birth cohort study. PLoS ONE 13(10):e0206160. 10.1371/journal.pone.0206160 30372455 10.1371/journal.pone.0206160 PMC6205641

[15] Zhang D, Ruchat SM, Silva-Jose C, Gil-Ares J, Barakat R, Sánchez-Polán M (2023) Influence of physical activity during pregnancy on type and duration of delivery, and epidural use: systematic review and meta-analysis. J Clin Med 12(15):5139. 10.3390/jcm12155139 37568541 10.3390/jcm12155139 PMC10419719

[16] Wiebe HW, Boulé NG, Chari R, Davenport MH (2015) The effect of supervised prenatal exercise on fetal growth: a meta-analysis. Obstet Gynecol 125(5):1185–1194. 10.1097/AOG.0000000000000801 25932847 10.1097/AOG.0000000000000801

[17] Harder T, Rodekamp E, Schellong K, Dudenhausen JW, Plagemann A (2007) Birth weight and subsequent risk of Type 2 Diabetes: a meta-analysis. Am J Epidemiol 165(8):849–857. 10.1093/aje/kwk071 17215379 10.1093/aje/kwk071

[18] Chen Y, Ma G, Hu Y, Yang Q, Deavila JM, Zhu MJ et al (2021) Effects of maternal exercise during pregnancy on perinatal growth and childhood obesity outcomes: a meta-analysis and meta-regression. Sports Med Auckl NZ 51(11):2329–2347. 10.1007/s40279-021-01499-6 10.1007/s40279-021-01499-6 34143412

[19] Cui H, Li H, Huang J, Wu Y, Wei Y, Li M (2025) The effect of exercise on the adverse neonatal outcomes related to women with gestational diabetes mellitus: a systematic review and meta-analysis. Front Clin Diabetes Healthc 6:1566577. 10.3389/fcdhc.2025.1566577 40235647 10.3389/fcdhc.2025.1566577 PMC11997568

[20] Hopkins SA, Baldi JC, Cutfield WS, McCowan L, Hofman PL (2010) Exercise training in pregnancy reduces offspring size without changes in maternal insulin sensitivity. J Clin Endocrinol Metab 95(5):2080–2088. 10.1210/jc.2009-2255 20335449 10.1210/jc.2009-2255

[21] Liu X, Guo X, Jie R, Tang Y (2025) The effects of high intensity exercise on pregnancy outcomes and complications during pregnancy: a meta-analysis of randomized controlled trials. Eur J Appl Physiol. 10.1007/s00421-025-05730-4 39976761 10.1007/s00421-025-05730-4

[22] McDonald SM, Newton E, Strickland D, Isler C, Haven K, Kelley G et al (2020) Influence of prenatal aerobic exercise on fetal morphometry. Matern Child Health J 24(11):1367–1375. 10.1007/s10995-020-03000-7 32833128 10.1007/s10995-020-03000-7 PMC12159129

[23] McDonald SM, Isler C, Haven K, Newton E, Kuehn D, Kelley G et al (2021) Moderate intensity aerobic exercise during pregnancy and 1-month infant Morphometry. Birth Defects Res 113(3):238–247. 10.1002/bdr2.1671 33522701 10.1002/bdr2.1671 PMC12162150

